# Rural–urban health-seeking behaviours for non-communicable diseases in Sierra Leone

**DOI:** 10.1136/bmjgh-2019-002024

**Published:** 2020-03-02

**Authors:** Ayesha Idriss, Karin Diaconu, Guanyang Zou, Reynold GB Senesi, Haja Wurie, Sophie Witter

**Affiliations:** 1 NIHR Research Unit of Health in Fragility, College of Medicine and Allied Health Sciences, University of Sierra Leone, Freetown, Sierra Leone; 2 NIHR Research Unit of Health in Fragility, Institute for Global Health and Development, Queen Margaret University Edinburgh, Musselburgh, UK; 3 School of Economics and Management, Guangzhou University of Chinese Medicine, Guangzhou, China, Guangzhou, China; 4 Non-communicable Diseases and Mental Health Directorate, Sierra Leone Ministry of Health and Sanitation, Freetown, Western Area, Sierra Leone

**Keywords:** health systems, cardiovascular disease, diabetes, hypertension, qualitative study

## Abstract

**Introduction:**

Non-communicable diseases (NCDs) are the leading cause of mortality globally. In Africa, they are expected to increase by 25% by 2030. However, very little is known about community perceptions of risk factors and factors influencing health-seeking behaviour, especially in fragile settings. Understanding these is critical to effectively address this epidemic, especially in low-resource settings.

**Methods:**

We use participatory group model building techniques to probe knowledge and perceptions of NCD conditions and their causes, health-seeking patterns for NCDs and factors affecting these health-seeking patterns. Our participants were 116 local leaders and community members in three sites in Western Area (urban) and Bombali District (rural), Sierra Leone. Data were analysed using a prior framework for NCD care seeking developed in Ghana.

**Results:**

Our findings suggest adequate basic knowledge of causes and symptoms of the common NCDs, in rural and urban areas, although there is a tendency to highlight and react to severe symptoms. Urban and rural communities have access to a complex network of formal and informal, traditional and biomedical, spiritual and secular health providers. We highlight multiple narratives of causal factors which community members can hold, and how these and social networks influence their care seeking. Care seeking is influenced by a number of factors, including supply-side factors (proximity and cost), previous experiences of care, disease-specific factors, such as acute presentation, and personal and community beliefs about the appropriateness of different strategies.

**Conclusion:**

This article adds to the limited literature on community understanding of NCDs and its associated health-seeking behaviour in fragile settings. It is important to further elucidate these factors, which power hybrid journeys including non-care seeking, failure to prevent and self-manage effectively, and considerable expenditure for households, in order to improve prevention and management of NCDs in fragile settings such as Sierra Leone.

**Key questions**

**What is already known?**

- There are few studies examining community knowledge of and care seeking for non-communicable diseases (NCDs) in Africa; those that exist have documented a range of strategies, including biomedical and spiritual, active and passive, as well as inaction.

**What are the new findings?**

- Via the case of Sierra Leone, we examine community knowledge of NCD causes in Sierra Leone, which is adequate in rural and urban settings for common conditions.
- However, causal narratives and care seeking are complex and influenced by supply-side factors (such as proximity to providers, cost and previous experiences of care), but also disease-specific factors (such as acute presentation) and sociocultural factors (such as personal and community beliefs about the appropriateness of different strategies).
- Care-seeking strategies include a combination of traditional and biomedical, formal and informal providers.

**What do the new findings imply?**

- Preventive strategies and better support for management of NCDs are urgent in these fragile settings and need to be informed by deeper understanding of community perceptions and links with existing community actors in order to effectively address this growing epidemic.

## Introduction

Non-communicable diseases (NCDs) are currently the leading cause of mortality globally^1 2^ and across the African region, where they are expected to increase by 25% by 2030.^3^ Africa’s chronic disease burden is attributed to many factors, including increased life expectancy and lifestyle changes, such as increasingly sedentary lifestyles and diets high in saturated fat, salt and sugar. These, in turn, are linked to wider structural factors such as industrialisation, urbanisation and increasing food market globalisation.^4^

Sierra Leone is a West African country with a population of over seven million,^5^ where 60% of the population lives below the poverty line. The gross domestic product per capita is $1651, and it is ranked number 14 in the top 25 poorest countries globally.^6^ The country, with its capital in Freetown, is made up of 5 administrative regions and 16 districts.^7^ It has one of the lowest life expectancies (52 years for men and 54 years for women) in the world.^8^ The health system in Sierra Leone is also one of the most fragile in the world, meaning that it has capacity gaps in relation to its ability and willingness to provide essential services for its population.^9^ This is partly due to the civil war between 1991 and 2002 that left thousands of people dead, much of the country's infrastructure (including health facilities) destroyed and millions of people displaced as refugees in neighbouring countries.^10^ The health system in Sierra Leone was further burdened by the Ebola virus disease outbreak in 2014, which further exposed the weakness in the existing health system and pointed to the need for holistic reconstruction. While the country is facing a high communicable disease burden, NCDs present an increasing burden for this already fragile health system,^11^ which has unfortunately received inadequate attention historically.

The WHO estimates the percentage of deaths attributable to NCDs in Sierra Leone to have increased from 18% in 2008 to 26% in 2012, with cardiovascular diseases, diabetes and cancer accounting for 9% and 2%, respectively.^12^ All other NCDs are reported to account for 12%. The prevalence of known risk factors is also increasing.^13^ The WHO 2009 STEPwise approach to surveillance (STEPS) in Sierra Leone established high prevalence of NCD risk factors among the adult population aged 25–64 years: 34% used tobacco; 17% used alcohol; around 90% consumed fewer than five servings of fruit/vegetables on average daily; 16% and 75% engaged in low and moderate levels of physical activity, respectively; and 31% lived sedentary lifestyles.^14^ The survey also showed that of the 4997 adults included in the STEPS survey, about 35% of both men and women had high blood pressure (BP) or were currently on medication for high BP, with over 90% of participants having a high BP but not on medication, highlighting a highly unmet need. In 2016, a study also reported that the prevalence of obese and overweight individuals was 8.7% and 27.7%, respectively, among adults.^12^ The staple food is rice served with meat, fish and vegetables, mainly cooked with palm oil.

A literature scoping review,^11^ WHO country reports^15^ and analysis of Sierra Leone’s readiness to tackle the rising NCD burden^16^ suggest that several health system-related challenges exist. First, the management of NCDs is not supported via dedicated funding mechanisms. This means that care for the general population suffering from chronic conditions is not free, though patients may sometimes benefit from receiving medicines and care donated by other organisations. Second, no NCD-specific national guidelines exist. While WHO initiatives, such as Package of Essential Noncommunicable Disease Interventions, have been trialled and integrated within the care offered in the country, this has not been done cohesively and gaps still exist, for example, in relation to cardiovascular disease prevention and management. Third, critical gaps within the healthcare system undermine coverage and quality of provision. For example, community health officers deliver health talks in relation to NCDs. However, lack of diagnostic equipment impairs their ability to diagnose, monitor and further refer such conditions. Historically, staff training has been undertaken by external organisations, with trainings frequently not quality assured and inconsistently offered.

In addition to system challenges, community challenges also abound. Research suggests that lay knowledge of the risk factors of diabetes and hypertension is poor.^17^ Affordability and access to health services—particularly in rural areas where health services sometimes lie beyond the reach of the people—are documented barriers to formal health service use and, coupled with culture and local values, are noted to result in high informal care use. A strong informal network of health providers, dominated by traditional healers, already exists within the country,^18^ and available evidence suggests that approximately 30% of persons have visited a traditional healer in Sierra Leone at least once.^19 20^ This is in line with wider evidence, which suggests that over 50% of persons in sub-Saharan Africa have sought care from traditional, complementary and complementary medicine providers.^21^

Communities play a key role in shaping not only health-seeking behaviours^22 23^ but also the lifestyle decisions that drive chronic illness onset and continuation, and people’s interpretations of the responses to pain and suffering. Culture and values play significant roles in decision making: cultural values influence patient preferences, and the status and reputation of providers in communities and trust in informal providers are all factors that influence whom people approach for medical care.^24^ Community-level norms and practices shape whether or not people will make best use of medical services when these are available, in terms of appropriate access and optimal adherence to medical advice.^25^ In addition, community networks mediate the diffusion of health-related knowledge from health professionals to vulnerable individuals.^26^ However, there is a dearth of evidence on this in the context of Sierra Leone.

We use a method called participatory systems dynamics to understand how chronic conditions are perceived and experienced and how they drive health-seeking practices, and to explore how they fit in a previously described framework (see box 1).

Box 1 Illness practices described in de-Graft Aikins 2005*

**Biomedical management is marked as follows:**

- Explanations on causes of diseases and solutions are predominantly biomedical.
- Doctors are recognised as diagnostic and pharmacological experts offering superior knowledge and practices over ethnomedical and faith healing systems, which are viewed as inept.

**Spiritual action is marked as follows:**

- Explanations of causes of disease predominantly refer to spiritual issues (eg, witchcraft and sorcery) and demand intervention from religious specialists.
- Persons may distinguish between legitimate (formal religious) versus illegitimate (traditional healing and traditional religious) practices as part of health seeking.

**Cure seeking refers to how people practise the above-mentioned two practices:**

- Active cure seeking refers to the continued need of persons to pursue a cure for their chronic condition, for example, by continuously seeing a multitude of biomedical and spiritual practitioners in the hope that a cure would materialise.
- Passive cure seeking, in contrast, refers to the intermittent consultation of biomedical and spiritual providers when chronic conditions exacerbate for short periods of time. This practice is enacted in the name of cure seeking time and time again in order to find psychological alleviation.

**Medical inaction refers to**

- ‘Passive withdrawal from drug and dietary management’ (p4) by those experiencing chronic suffering, exacerbating conditions, limited family support and limited financial ability to seek care.
- May still involve spiritual action but directed towards seeking ‘death as an end to chronic suffering and a miracle’ (p5).

*Summary developed by authors.

## Methods

### Study design

Given the increased recognition that health and community systems operate as complex adaptive systems,^27 28^ we conducted participatory group model building sessions with both health system actors (national policy makers and district health representatives and urban and rural healthcare providers) and communities (urban and rural community leaders and members). We focus here only on community-related work and present all work pertaining to health system elements elsewhere^16^). Development of research questions and design of the study were informed by findings from a scoping review that was done earlier to gain an understanding of the priority areas of research for NCDs.

### Study site selection

The study sites chosen were contrasting settings: urban (Freetown, which is the capital city of Sierra Leone and is in the Western Area district) and rural (Makeni, the provincial head quarter town of Bombali District in northern Sierra Leone and its surrounding villages). A sociocultural line divides the urban and rural areas of Sierra Leone. Urban areas are mostly influenced by Western culture and rural areas by traditional practices. Poverty levels are higher in rural areas than in urban areas. People living in urban areas have better access to public services, including health, education and employment.

### Participant sampling and recruitment

Participants were sampled purposively, selecting specific experiences and/or roles and functions performed within the local community. For example, we targeted community leadership—religious leaders, teachers and local politicians within communities—as they are likely to shape public opinion generally and specifically in relation to NCDs. For community members, we focused on persons who were either healthy but interested in the topic of study, were at risk of developing NCD or were acting as caretakers of persons with such illnesses. Convenience and snow-ball sampling techniques were employed to ensure the recruitment of relevant participants. During each field visit, recruitment took place 1–2 days prior to the research team’s visit to a specific location. Research team members contacted local healthcare staff and local leaders and, based on their recommendations and mobilisation, further contacted local community members (either via phone or word of mouth) and invited them to the relevant group model building session. A breakdown of the number of study participants (116 in total: 69 urban and 47 rural) is given in table 1.

**Table 1:** Group model building session characteristics

Setting type	Number and type of group model building session	Workshop format	Participants involved	Topics discussed
Urban	One session for local leaders	One group only (no gender split)	21 local leaders (tribal chiefs, local politicians, counsel men/women, traditional healers, religious leaders, teachers and association representatives (youths)), of which 12 were male and 9 were female	Knowledge and perceptions of NCD conditions and their causes; health-seeking patterns for NCD-related conditions, including exploration of what ‘care’ consists of at different points; factors affecting health-seeking patterns, including their relative priority to participants
	One session for local community, informal settlement	Groups split by gender	26 community members interested in NCD service delivery, caregivers of family members with conditions or at risk of developing conditions, of which 13 were male and 13 were female	
	One session for local community	One group only (no gender split)	22 community members interested in NCD service delivery, caregivers of family members with conditions or at risk of developing conditions, of which 13 were male and 9 were female	
Rural	One session for local leaders	Groups split by gender	24 local leaders (tribal chiefs, local politicians, counsel men/women, traditional healers, religious leaders, teachers and association representatives (youths)), of which 16 were male and 8 were female	
	One session for local community	Groups split by gender	23 community members interested in NCD service delivery, caregivers of family members with conditions or at risk of developing conditions, of which 10 were male and 13 were female	

NCD, non-communicable disease.

### Group model building sessions

Table 1 summarises the number of sessions conducted, the number of participants involved and the topics under discussion across sessions. Each group model building session was hosted in a location convenient to participants. The research team aimed to host sessions in locations separate from local healthcare facilities to minimise any potential bias for participants. While this was not always possible, in those community sessions conducted at health facilities, the research team carefully moderated the role of healthcare providers on the premises: staff were told not to take part and were encouraged to proceed with normal duties elsewhere. As we had anticipated that gender-related differences in care seeking were likely, we aimed to split participants within each session by gender (see table 1); this was not possible in cases where under three participants of one gender were attending.

The research team started each group model building session by explaining the purpose of the project, informing participants that their presence and participation was voluntary and securing oral consent. The sessions lasted 4.5 hours on average; possible groups were split by gender; and activities were tailored to the specific topics to be discussed and group of participants involved. Each activity had a specific ‘script’ outlining the task and objectives at hand; appendix 1 is a protocol of the activities conducted and illustrates the types of group model building scripts used. Scripts were elaborated and refined by the research group and the majority draw on publicly available scripts (Scriptopaedia) and research protocols on the study of social connections. For the purposes of the study, new scripts relating specifically to the identification of ‘points of fragility’ were elaborated. For each group model building session, field notes summarising discussions and highlighting interesting findings and emerging hypotheses on health-seeking dynamics were kept by a group of observers. Where possible, observers captured participant quotes as well.

Group model building sessions resulted in a number of ‘rich pictures’ representing depictions elaborated by study participants relating to their perceptions of NCD causes, manifestations and health-seeking actions. Participants were further asked to reflect on the rich pictures and to identify key variables which contribute to NCD onset and shape health seeking; ultimately, participants used and linked these variables within incipient causal loop models.

### Analysis

The causal loop models resulting from the various group model building workshops were iteratively refined by the research team: on consultation of field notes, variables were reworded for clarity, and pathways discussed by participants were further elaborated. In addition, models from different sites were compared to identify differences in community dynamics due to setting-specific characteristics; that is, we considered whether participants of different genders, ages or roles within the community described different variables or pathways. Where no such differences existed, models were combined; where differences were present but model consolidation was possible, differences were indicated by colour-coding variables or pathways on diagrams and adding explanatory notes. Third, researchers discussed the loops driving community health-seeking behaviours, and once descriptive findings were firmed up, we proceeded to compare these to the theoretical framework of de-Graft Aikins.^29^

### Patient and public involvement

Patients were not involved in this study but rather the public. We consulted local leaders prior to data collection within their different communities. Participants were not directly involved in the development of research questions, design of the study and recruitment to and conduct of the study.

We engaged relevant NCD stakeholders, community representatives and civil society activists to share and discuss the findings of the scoping review. Afterwards, we designed this study, which elicited stakeholder’s, community representatives’ and civil society activists’ perceptions.

## Results

We conducted three urban group model building sessions involving 69 participants (21 local leaders, 26 informal settlement-dwelling community members and 22 local community dwellers) and two rural sessions involving 47 participants (24 local leaders and 23 rural community members). Where possible, workshops split participants according to gender in order to elaborate gender-specific accounts of health seeking (see table 1).

We structure our presentation of findings along the broad illness practices and health-seeking behaviours identified by de-Graft Aikins in Ghana (see box 1) and in a further section offer reflections on divergent and convergent findings.

### Biomedical management

In both rural and urban communities, chronic conditions were largely recognised and framed as a biomedical problem. Participants were generally able to recognise what a chronic disease was and gave examples of hypertension and diabetes, as well as cancers, most frequently. However, more diseases were recognised and named in urban settings (eg, urban community leaders and members noted diabetes, hypertension, cancer, asthma and epilepsy), while rural counterparts mainly recognised diabetes and hypertension. Two themes stood out in relation to communities’ perceptions of diseases and their attribution to either biomedical or other causes.

#### Communities associate chronic conditions primarily with severe events or complications

**Figure 1:**
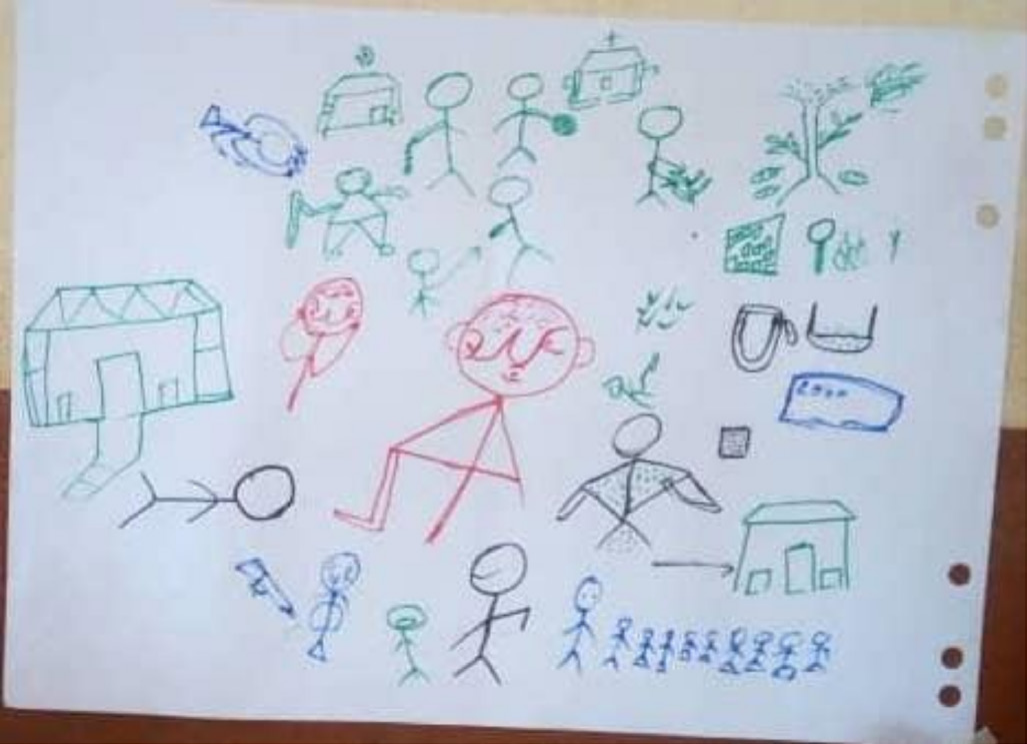
Rich picture of participants’ knowledge and perceptions of non-communicable disease conditions (in red), their causes (in blue and black) and the places they go to seek help (in green).

When asked to draw what they perceive as chronic conditions, most of the drawings were of disease complications or severe events (see figure 1); de-Graft Aikins notes these as episodes where chronic conditions get worse. For example, participants drew people who had experienced a stroke or were affected by leg ulcers. Nevertheless, knowledge on primary symptoms (eg, obesity, frequent urination, headaches, fatigue and weight loss) was also present but emphasised less than more severe symptoms.

Commonly, diabetes was referred to as ‘too much sugar and carbohydrate’; only one participant understood it to be a dysfunction of the pancreas. Hypertension, on the other hand, was mostly understood to be caused by ‘too much salt in the diet, too much thinking about family problems or worrying about being able to provide daily meals, which can make you crazy and thus stressed out’.

#### Biomedical risks are recognised alongside wider social determinants

Participants associated the development of chronic diseases, especially of diabetes and hypertension, with unhealthy diets. Participants noted the consumption of too much oil, sugar, salt, alcohol and starchy foods as potential risk factors and linked these to obesity, fatigue and illness; similarly, participants recognised and spoke of the negative role tobacco played in their community and on their health. Rural community members also noted that disease sometimes just manifests randomly, though some noted that it may be hereditary, thus hinting at genetic predisposition. Urban community members additionally spoke of lack of exercise—‘sitting too long’—as a risky behaviour which may increase the likelihood of disease onset.

Stress was also mentioned to cause hypertension and was described as a result of either poverty and not having enough money to look after the family or having to deal with too many wives and children, leading to anger and frustration. Cancer was perceived to be caused by tobacco and alcohol use.

Social issues like poverty and urbanisation (including, eg, greater imports of processed foods into the country and general diet changes due to product availability), westernisation of foods (eg, the availability of processed or fried fatty foods) and changes in occupation (moving away from manual and physical labour towards office-based jobs) were widely linked with the onset of biomedical risk factors. For example, across both urban and rural groups, community leaders reflected that persons experience high levels of stress due to financial insecurity and the need to provide for large families.

…people [are] used to eating natural foods; these days I don’t know what am eating! I am just eating!

…some old people (70–80 year-old) are strong because they eat natural food.

#### Seeking biomedical help is not restricted to formal public medical institutions

**Figure 2:**
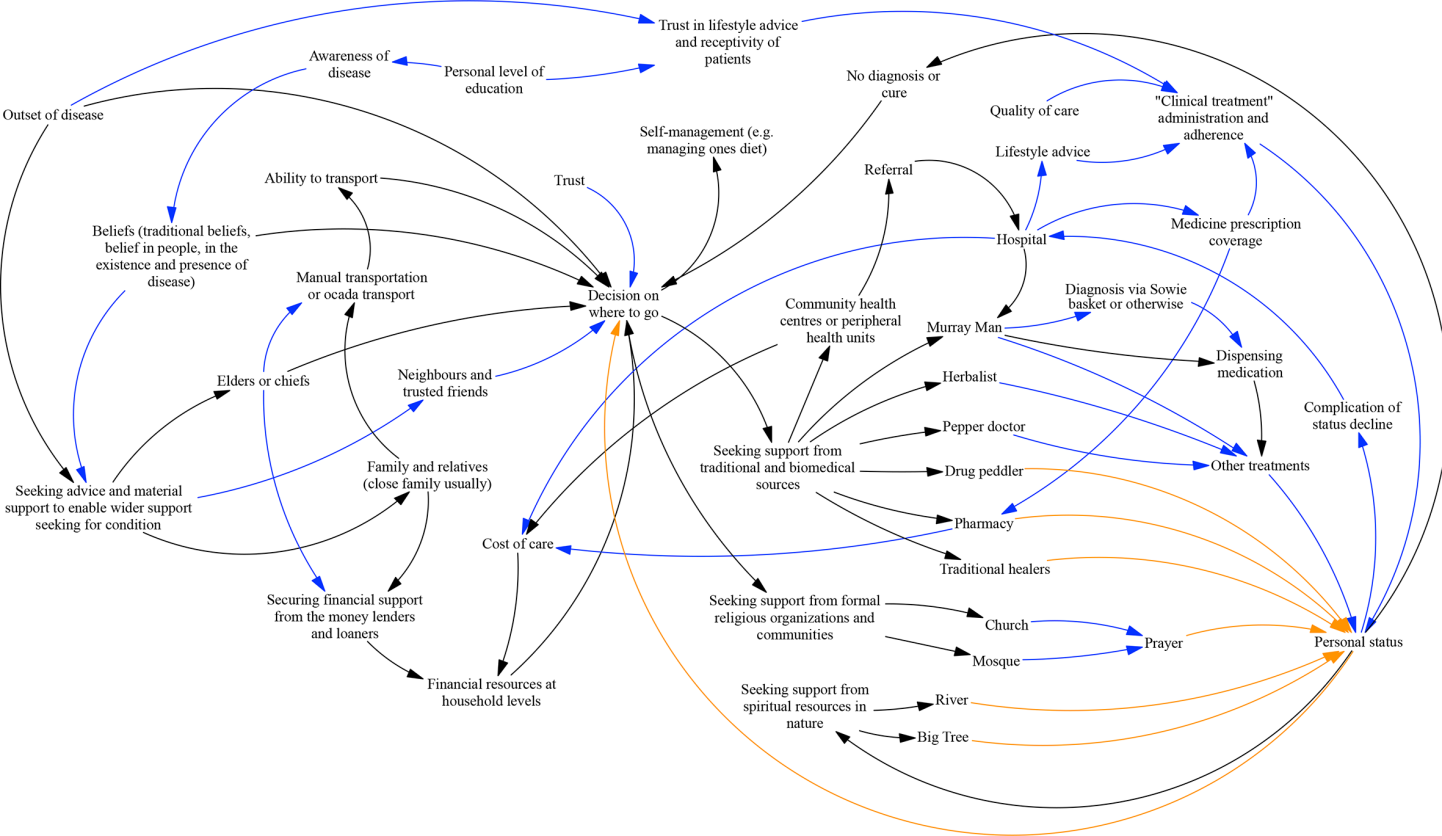
An example of cure seeking patterns for non-communicable diseases and factors affecting these cure seeking patterns in the rural setting. Black arrows for the men; blue arrows for links added/elaborated additionally by women and orange arrows added in by the research team for completeness (based on discussions of participants.

Across both urban and rural communities, participants largely discussed health seeking in relation to persons affected by chronic disease-related complications. When severe complications occur, the health-seeking journey begins and people seek advice, companionship and counselling support from family and friends (see figure 2). Based on this advice, a decision on where to go is made, which may involve public health institutions, providers or hospitals, churches and mosques or religious leaders, shrines, oracles and idols to seek health-related support and care.

Most commonly, people reported health-seeking practices which aimed at managing symptoms or preventing further symptom exacerbation (eg, in relation to leg ulcers). This meant seeking pharmacological care from informal drug peddlers in the local community. These providers were noted to dispense medicines and other pharmaceuticals within their routine practice despite not being recognised officially by the health system and thus practising out with formal laws. Herbalists, another type of informal traditional health provider, were recognised to fulfil a similar role.

Participants also noted seeking care from formal health providers. This avenue of health seeking was more frequently described by male participants; however, participants of both genders agreed that the formal health system was often accessed only as a last resort once other informal and traditional providers had been sought. When prompted to reflect on the quality of care received at the different points of call (as identified in figure 2), participants agreed that formal health institutions, that is, clinics and hospitals, provided higher quality care than informal and traditional providers. Participants additionally noted that pharmacological treatments were not only restricted to persons or institutions recognised as health providers but also practised by those engaged in offering spiritual care (eg, herbalists; see table 2).

**Table 2:** Example of spiritual and traditional providers active in Sierra Leone and their practices and functions

Type of spiritual and traditional provider	Provider practice	Function
Pepper doctor	Offers medication for condition	Principally offers biomedical treatment
Herbalist	Offers diagnosis of the condition and herbal-based treatments (eg, lotions)	Limited biomedical element (eg, lotion) and distinguishable diagnostic and treatment step, traditional healer
Moray man and/or alpha man	Diagnosis includes religious elements of care, recitation of incantations (eg, alpha man uses Koran during practice) and additionally may offer non-conventional ‘medicine’ or potential referrals to other providers	Moderate biomedical element of care and distinguishable diagnosis, treatment and referral steps; spiritual and traditional healer
Church or mosque	Offer prayer and gives you blessed water and oil to rub	No biomedical elements, completely spiritual
Yabai woman (soothsayer)	Consults the person and determines who cast a spell on them, bewitches the spell-caster	No biomedical elements, completely spiritual

### Spiritual action

#### While chronic conditions are recognised as primarily biomedical problems, spiritual components continue to be recognised and shape health seeking

While the majority of participants attributed the emergence of chronic diseases to biomedical problems, including as influenced by societal change (see previous section), participants still noted that Sierra Leoneans hold entrenched beliefs which imply disease onset to be associated with spiritual causes such as evil spirits or witchcraft. Such views were more strongly articulated in rural settings but were present across urban communities (both informal settlement and local community dwellings) as well.

Across all groups, when probed in relation to health-seeking practices, participants noted seeking care from spiritual and/traditional sources such as spiritualists (locally known ‘Moray man’ and ‘alpha man’) immediately after identifying informal drug peddlers and herbalists as a first point of call. On further probing, a set of distinct providers offering increasingly spiritual-themed care emerged from participant accounts (see figure 2 and table 2).

Participants noted that during episodes of extreme suffering or disease exacerbation, those persons holding strong spiritual beliefs in their communities would turn to traditional providers which incorporate spiritual elements of care. For example, for a diabetic patient with a wound that does not heal or a patient who develops retinopathy, a spell being cast or witchcraft may be mentioned as the cause. To seek care for this, one may consult a ‘Yabai’ woman (see table 2), who could bewitch the spell caster, thus reversing the original spell and leading to alleviation of symptoms.

Overall, participants across all groups agreed that seeking care from traditional providers as those listed in table 2 is unlikely to result in health status improvements and that as a result, they would eventually have to seek care from the formal health system as well. However, the health-seeking journey up to this point involves multiple consultations with informal health providers and traditional providers.

### Cure seeking and medical inaction

#### Community members’ views on chronic disease strongly inform their fragmented help-seeking patterns and cure-seeking

Most of the time, participants discussed chronic diseases in relation to management of symptoms or discrete serious events corresponding to severe complications (eg, stroke). There was limited discussion of problems being chronic and persons generally reported approaching health seeking and management of the disease according to the severity of the issue, hoping for quick alleviation of symptoms or a cure. When symptoms are minor, participants noted being able to manage the problem locally with drug peddlers, pharmacists or self-management (eg, some participants just ‘drink some water and rest’).

…we go to a particular tree or rock for prayers.

…some read the Quran or Bible at home or go to churches or mosques for prayers.

…some go to the rivers to seek help from the river devil - mamy water [mermaid]; water goddess.

In the case of a clear complication of the disease with major symptoms, persons reported seeking care from both formal health system and informal providers, such as herbalists, religious leaders, Moray men, alpha men, or elements of nature, such as rivers, trees and rocks. When probed to identify influences on health seeking, participants identified trust in providers, immediacy (ie, local availability, accessibility and affordability) and beliefs as major influences. Once identifying these factors in a causal loop diagram (see figure 2) and on reflecting on issues of quality of care, participants looked at their models and they themselves said it looked chaotic. However, it was unclear whether any behaviour change would then occur.

#### Self-management of conditions can be both active and/or result in medical inaction

Some community members (those with higher levels of education) noted that behavioural change was possible once persons truly realised that their disease condition was chronic. For example, a teacher in a rural community we visited narrated how an initial diagnosis of hypertension and lifestyle counselling received from a formal health provider made him realise that it would be less costly and more effective to change his salt consumption than it would be to continue seeking care for episodes of fainting and injury at clinics.

However, some participants noted that despite not having a formal diagnosis, they were able to recognise having a chronic illness. In this case, it was perceived that a formal diagnosis would not change much but would add to their general level of worry and suffering. Largely, participants describing this situation were the elders in the community and also those most affected by severe chronic disease complications (stroke, glaucoma and amputation). They noted that they had lived through severe events in Sierra Leone’s history and that they did not seek help from anyone and kept their problems to themselves.

## Discussion

Our study into the community dynamics surrounding NCDs in Sierra Leone reveals that both urban and rural communities have a working knowledge of chronic conditions and symptoms but associate these mainly with severe complications. While urban communities were able to name more disease examples, both communities were able to identify genetic and lifestyle-related chronic disease risk factors as well as discuss these more widely in terms of social determinants, such as poverty, urbanisation and globalisation.

We document that both urban and rural communities have access to a complex network of formal, informal and traditional health providers, able to offer a wide and mixed range of biomedical and spiritual support, as well as diagnostic and treatment services for chronic diseases. Health seeking is shaped not only by availability of providers but also by the social network and capital of those affected by the disease, their trust in care providers, the immediacy of accessing them (availability, affordability and accessibility) and beliefs on disease causes and sociocultural factors. Perceptions on quality of care, as distilled via wider social networks or even from personal experience, add to the complexity of health seeking. Our study highlights that health seeking from formal health institutions is often the last resort and accessed largely once other options are exhausted. This finding is matched by our analysis of the health system, which faces severe limitations in terms of funding, medicines, equipment and training to be able to meet growing NCD needs.^16^

We attempted to present findings according to the de-Graft Aikins framework, which identifies four predominant health seeking (or so called ‘healer shopping’) practices: biomedical management, spiritual action, cure seeking and medical inaction (see box 1). Our findings were largely concordant with de-Graft Aikin’s findings in Ghana, particularly in relation to passive care seeking; however, we note two areas of potential enhancement to the way the framework may be used.

First, given a broader focus on health seeking, participants of our workshops additionally discussed the influence of their social and community networks on health-seeking practices.

Across all groups, families were noted as a key point of call when first considering which provider to approach and, in the case of rural women in particular, friends and neighbours were also highlighted as helpful sources of information and advice. Participants, and in particular men, noted that their social network and potentially their local leaders further played a critical part in helping raise the necessary funds (or capital in the case of rural residents (eg, livestock)) to engage in health-seeking behaviours. The health-seeking preferences of those family, neighbourhood and local leadership networks were noted to influence which health-seeking pathway persons chose (eg, some rural leaders noted they were likelier to loan money for seeking care from health providers). Observers of workshop discussions noted that local pharmacists, drug peddlers and pepper doctors were described as not only the most easily accessible but also the most uncontroversial persons to be approached in health seeking. Understanding and expanding de Graft’s framework to also consider how social networks and capital shape health-seeking practices is therefore of particular value when considering, for example, interventions that wish to shape the broader health-seeking landscape.

Second, we note that it is necessary to emphasise that several of the health-seeking practices de Graft identifies may overlap, specifically, for example, the concepts of biomedical and spiritual illness practices. On first reading of de-Graft Aikin’s work, one may be tempted to view biomedical and spiritual practices as internally consistent behaviours; that is, those persons attributing chronic disease development to biomedical factors would be expected to seek care from biomedical providers. In contexts like Sierra Leone, and specifically in our workshops, we have noted that some persons did indeed offer internally consistent accounts. However, our findings also indicated that the majority of persons had a predominantly biomedical understanding of chronic conditions which coexisted with spiritual explanations of disease. Similarly, in this context, providers cannot be easily categorised into biomedical or spiritual (see table 2), and instead, a large number of providers offer hybrid spiritual–biomedical care. Acknowledging that health-seeking practices may therefore overlap, which has consequences for how NCDs are managed when multiple providers are consulted, is critical to securing health outcomes.

We identify two areas where the de-Graft Aikins framework could evolve in light of our findings. First, consideration of social networks and capital is critical to understanding health seeking and therefore should be added as a framework element. While health seeking has come under critique as a ‘thin concept’, proponents of more nuanced approaches suggest that issues of social capital and networks are instrumental in understanding how populations engage with health systems.^23^ Second, we note the distinction between biomedical versus spiritual practice is not clear-cut, and as such, researchers should seek to explore this along a gradient (including in identifying the types of services and wider support offered by different types of providers), in conjunction with in-depth analysis as already proposed by de-Graft Aikins on disease explanations. In our case, very few practitioners clearly offered and practised only one type of service, while several other providers combined practices, and this suggests a more complex picture emerging in relation to NCDs in Sierra Leone. An important next step for research would be to use the analysis in this article to develop effective engagement strategies with different community actors and to test their effectiveness in addressing the urgent need for more effective prevention and management of common NCDs in rural and urban settings in Sierra Leone.

### Study limitations

We note a number of limitations. First, while our sampling strategy was purposive and our sample was sufficiently large, we conducted only a small set of workshops and focused on those with an interest in NCDs, such as community leaders and affected household members, who may therefore not be representative of the entire community. We made every attempt to visit settings which were representative of typical locations in Sierra Leone (rural, urban–informal settlements and urban–local communities). However, note that our urban setting was the capital itself and is therefore likely to be atypical of other urban settings in the country. Second, where possible and a large number of participants from both genders were available, we separated participants into groups by gender; this was not always possible, so it was not directly possible in some cases to compare urban–rural workshop outputs by gender grouping. Third, while participants elaborated a first version, the causal loop model presented here, it was not possible to go back to the same communities for validation. However, field notes and observations were used to triangulate and, as necessary, complete models.

## Conclusion

This article adds to the very limited literature on community understanding of NCDs and health-seeking behaviour in response to them in Africa. Using participatory group model-building methods in urban and rural settings in Sierra Leone, we highlight the multiple narratives of causal factors which community members can hold and how these and social networks influence their care seeking. Care seeking is influenced by a wide range of factors, including supply-side factors, such as proximity and cost and previous experiences of care, but also disease-specific factors, such as acute presentation, and personal and community beliefs about the appropriateness of different strategies. It is important to deepen our understanding of these factors, which power hybrid journeys, including non-care seeking, failure to prevent and self-manage effectively, and considerable expenditure for households, in order to improve prevention and management of NCDs in fragile and low-resource settings such as Sierra Leone.

## References

[1] Wild S, Roglic G, Green A, et al Global prevalence of diabetes: estimates for the year 2000 and projections for 2030. Diabetes Care 2004;27:1047–53. 10.2337/diacare.27.5.1047 15111519

[2] Boutayeb A The double burden of communicable and non-communicable diseases in developing countries. Trans R Soc Trop Med Hyg 2006;100:191–9. 10.1016/j.trstmh.2005.07.021 16274715

[3] Islam SMS, Purnat TD, Phuong NTA, et al Non-communicable diseases (NCDs) in developing countries: a symposium report. Global Health 2014;10:81 10.1186/s12992-014-0081-9 25498459 PMC4267750

[4] WHO, FAO Diet, nutrition and the prevention of chronic diseases. Report of a joint WHO/FAO expert consultation. Volume 916. World Health Organ Technical Report Series, 2003: 160. 12768890

[5] Statistics Sierra Leone. Statistics 2017.

[6] Trosclair E 10 Poverty facts about Sierra Leone [Internet], 2017 Available: https://www.borgenmagazine.com/facts-poverty-in-sierra-leone/ [Accessed 29 Nov 2019].

[7] Cham K Sierra Leone unveils new geographical map - Africa Review. Africa Review, 2017.

[8] World Health Organization WHO | Sierra Leone [Internet], 2016 Available: http://www.who.int/countries/sle/en/

[9] McPake B, Witter S, Ssali S, et al Ebola in the context of conflict affected states and health systems: case studies of northern Uganda and Sierra Leone. Confl Health 2015;9:1–9. 10.1186/s13031-015-0052-7 26257823 PMC4529686

[10] Mackinnon J, Maclaren B Human Resources for health challenges in fragile states : Evidence from Sierra Leone, South Sudan and Zimbabwe. J Heal Econ 2012;1:1–18.

[11] Idriss A, Wurie HR, Bertone M Kelly Elimian NV and MS. a scoping study on non-communicable diseases (NCDS) in Sierra Leone, 2018.

[12] World Health Organization Atlas of the African health statistics, 2017.

[13] World Health Organization The prevalence of the common risk factors of non-communicable diseases in Sierra Leone, 2009.

[14] Sierra Leone steps survey 2009. The prevalence of the common risk factors of non- communicable diseases in Sierra Leone.

[15] World Health Organization Noncomunicable diseases: Sierra Leone country profile, 2014.

[16] Witter S, Zou G, Karin D, et al Opportunities and challenges for delivering non-communicable disease prevention and services in a fragile setting: perceptions of policy-makers and health providers in Sierra Leone. Health Policy Plan 2019. 10.1186/s13031-019-0248-3 PMC6945746 31921333

[17] Mensah GA Epidemiology of stroke and high blood pressure in Africa. Heart 2008;94:697–705. 10.1136/hrt.2007.127753 18308869

[18] Irish Aid, United Kingdom Mapping Sierra Leone’s plural health system and how people navigate it, 2014: 1–4.

[19] Bakshi SS, McMahon S, George A, et al The role of traditional treatment on health care seeking by caregivers for sick children in Sierra Leone: results of a baseline survey. Acta Trop 2013;127:46–52. 10.1016/j.actatropica.2013.03.010 23545128

[20] Tomison T Working paper series 2013 Health-seeking behaviour and strategic healthcare planning in Sierra Leone. Int Dev 2013;44:13–139.

[21] James PB, Wardle J, Steel A, et al Traditional, complementary and alternative medicine use in sub-Saharan Africa: a systematic review. BMJ Glob Health 2018;3:e000895 10.1136/bmjgh-2018-000895 PMC6231111 30483405

[22] Ward H, Mertens T, Thomas C Health seeking behavior and the control of sexually transmitted diseases. Health policy and planning. Health Policy Plan 1997;12:19–28. 10166099 10.1093/heapol/12.1.19

[23] Mackian S A review of health seeking behaviour : problems and prospects. Heal Syst Dev 2003;27.

[24] Sudhinaraset M, Ingram M, Lofthouse HK, et al What is the role of informal healthcare providers in developing countries? A systematic review. PLoS One 2013;8:e54978 10.1371/journal.pone.0054978 23405101 PMC3566158

[25] de-Graft Aikins A, Unwin N, Agyemang C, et al Tackling Africa's chronic disease burden: from the local to the global. Global Health 2010;6:5 10.1186/1744-8603-6-5 20403167 PMC2873934

[26] Campbell C Contextualising health promotion within local community networks : Baron S, Fields J, Schuller T, Social capital and health. Oxford: Oxford University Press, 2001: 182–96.

[27] Hovmand PS Group model building and community-based system dynamics process : Hovmand PS, Community based system dynamics. New York, NY: Springer, 2014: 17–30.

[28] Barasa E, Mbau R, Gilson L What is resilience and how can it be Nurtured? A systematic review of empirical literature on organizational resilience. Int J Health Policy Manag 2018;7:491–503. 10.15171/ijhpm.2018.06 29935126 PMC6015506

[29] de-Graft Aikins A Healer Shopping in Africa: new evidence from rural-urban qualitative study of Ghanaian diabetes experiences. BMJ 2005;331:737–0. 10.1136/bmj.331.7519.737 16195290 PMC1239976

